# Heterozygous truncating variant of *TAOK1* in a boy with periventricular nodular heterotopia: a case report and literature review of *TAOK1*-related neurodevelopmental disorders

**DOI:** 10.1186/s12920-024-01840-8

**Published:** 2024-03-05

**Authors:** Anna Cavalli, Stefano Giuseppe Caraffi, Susanna Rizzi, Gabriele Trimarchi, Manuela Napoli, Daniele Frattini, Carlotta Spagnoli, Livia Garavelli, Carlo Fusco

**Affiliations:** 1 Child Neurology and Psychiatry Unit, Dipartimento Materno-Infantile, Arcispedale Santa Maria Nuova, Azienda USL-IRCCS di Reggio Emilia, 42123 Reggio Emilia, Italy; 2 Medical Genetics Unit, Dipartimento Materno-Infantile, Arcispedale Santa Maria Nuova, Azienda USL-IRCCS di Reggio Emilia, 42123 Reggio Emilia, Italy; 3 Neuroradiology Unit, Arcispedale santa Maria Nuova, Azienda USL-IRCCS di Reggio Emilia, 42123 Reggio Emilia, Italy

**Keywords:** *TAOK1*, Periventricular nodular heterotopia, PVNH, PNH, Neuronal migration disorders, Macrocephaly, Autism, ASD, Neurodevelopmental disorders

## Abstract

**Background:**

Thousand and one amino-acid kinase 1 (*TAOK1*) encodes the MAP3K protein kinase TAO1, which has recently been displayed to be essential for neuronal maturation and cortical differentiation during early brain development. Heterozygous variants in *TAOK1* have been reported in children with neurodevelopmental disorders, with or without macrocephaly, hypotonia and mild dysmorphic traits. Literature reports lack evidence of neuronal migration disorders in *TAOK1* patients, although studies in animal models suggest this possibility.

**Case presentation:**

We provide a clinical description of a child with a neurodevelopmental disorder due to a novel *TAOK1* truncating variant, whose brain magnetic resonance imaging displays periventricular nodular heterotopia.

**Conclusions:**

To our knowledge, this is the first report of a neuronal migration disorder in a patient with a *TAOK1*-related neurodevelopmental disorder, thus supporting the hypothesized pathogenic mechanisms of *TAOK1* defects.

**Supplementary Information:**

The online version contains supplementary material available at 10.1186/s12920-024-01840-8.

## Background

The advent of whole exome sequencing (WES) as a diagnostic tool has led to an overall diagnostic yield for unexplained neurodevelopmental disorders (NDDs) around 36%, and up to 53% for syndromic NDDs. WES has also led to a rapid increase in the identification of novel disease genes [1, 2].

The *TAOK1* gene on chromosome 17 (OMIM * 610,266) encodes the “thousand and one amino acid kinase 1” (TAO1), a ubiquitous serine/threonine protein kinase with a high expression level in brain [3]. It is part of a family of three kinases, TAOKs1-3, which are implicated in critical processes during neurogenesis such as stress-activated MAPK pathway, DNA damage response and regulation of microtubule stability and cytoskeleton dynamics [3–8].

Although large–scale WES and array-CGH studies had already identified *TAOK1* as a candidate gene for NDDs since 2011 [9, 10], the direct association has been confirmed only recently [11, 12]. Some of these studies clearly documented that defects in *TAOK1* expression can affect neuronal maturation and cortical development both in vitro and in animal models [4, 11, 13, 14].

To date, a few case reports and case series present short clinical descriptions of a total of 37 children with NDDs due to heterozygous *TAOK1* variants or genomic deletions encompassing the *TAOK1* region [11–16]. The phenotype consists of a spectrum of nonspecific overlapping clinical features, mainly represented by variable developmental delay with or without macrocephaly, autism spectrum disorder (ASD), hypotonia and mild dysmorphic traits. Most variants occurred *de novo*, but some were inherited by an affected or mildly affected parent, showing intrafamilial variability [14, 16].

To date, the reported data on brain magnetic resonance imaging (MRI) findings in *TAOK1*-related patients are scarce and non-specific [11, 16–18].

### Case presentation

The patient is a 5-year-old boy of Georgian origin, who was born at term by cesarean section (due to previous caesarean deliveries) from unrelated parents. Pregnancy and perinatal period were unremarkable. At birth, his weight was 4.185 g (+ 1.6 SD), length was 51 cm (-0.2 SD) and head circumference was 36 cm (+ 1 SD).

**Fig. 1:**
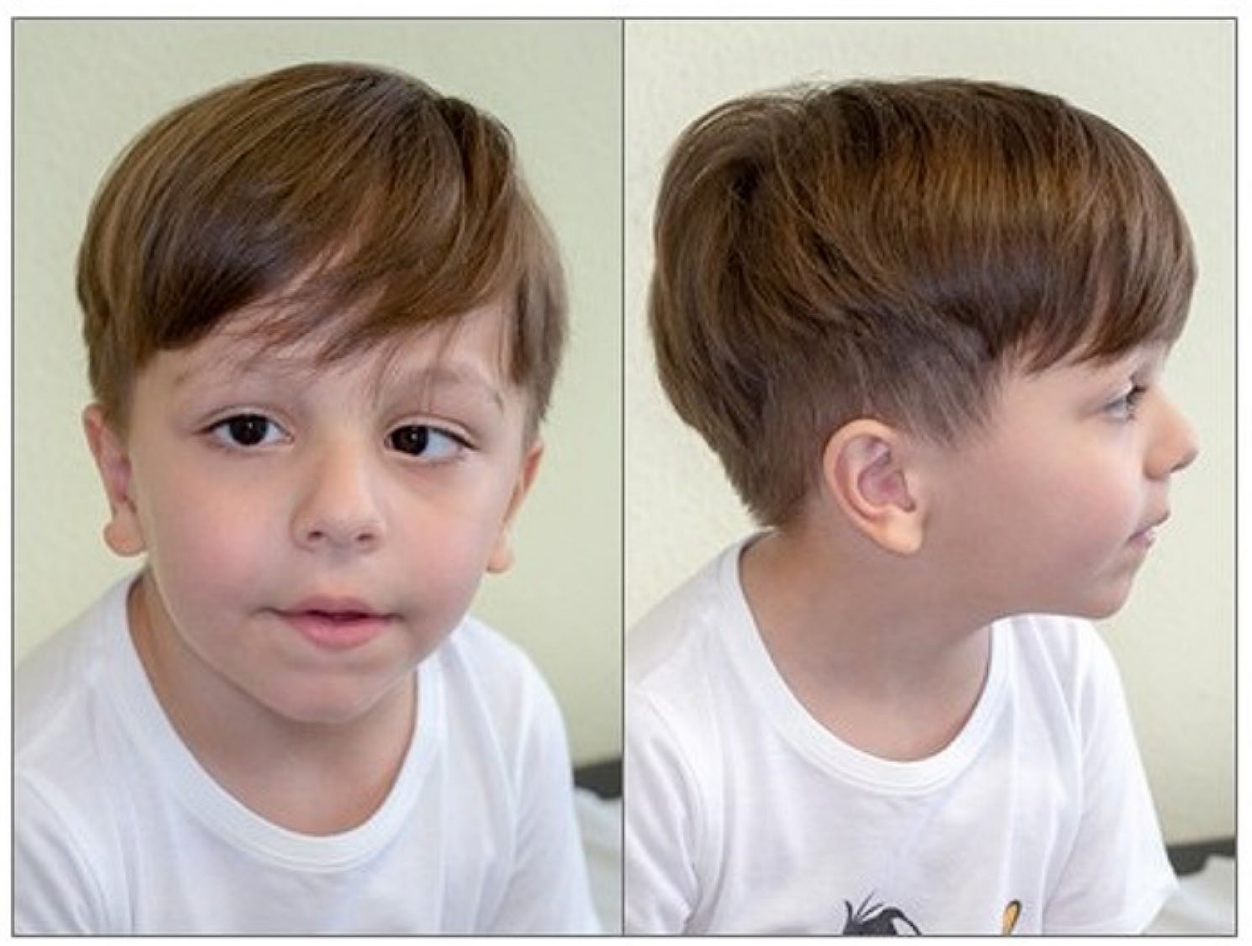
our patient’s phenotype showing macrocephaly and mildly dysmorphic traits (high forehead, long and pronounced philtrum, bulbous nose, uplifted earlobes, acquired left-eye esophoria)

During infancy linear growth and weight gain were normal, while head circumference increased at an abnormal rate that resulted in acquired macrocephaly. At the age of five years, body weight was 21.5 Kg (0 SD), height was 112.5 cm (+ 0.7 SD) and head circumference was 57 cm (+ 4.2 SD). He showed mild dysmorphic traits: high forehead, long and pronounced philtrum, bulbous nose and uplifted earlobes. Acquired left-eye esophoria appeared at the age of four years [Fig. 1].

Early psychomotor development appeared normal until the age of 18 months, when language stagnation with poor communicative purpose and hyperactive behaviour emerged. He also displayed poor eye contact during social interactions and selective eating. At the age of two years ASD was suspected by clinicians. At the age of five years, his language consisted of fewer than 20 disyllabic words. Bowel and bladder control were acquired almost completely and his eating was still selective. Motor skills were achieved within the normal ranges. Neither paroxysmal events nor seizures were reported; awake and sleep electroencephalograms were unremarkable.

At the age of four years, he underwent a comprehensive assessment with ADOS-2 Module 1 *(Autism Diagnostic Observation Schedule-Second Edition*), CARS-2 (*Childhood Autism Rating Scale, 2nd Edition)* and ADI-R (*Autism Diagnostic Interview-Revised*). He did not fully meet the criteria for autism diagnosis. His intelligence was in the normal range, with a nonverbal IQ of 98 (*Leiter − 3* scale). Abdomen ultrasound and audiometry were unremarkable.

**Fig. 2:**
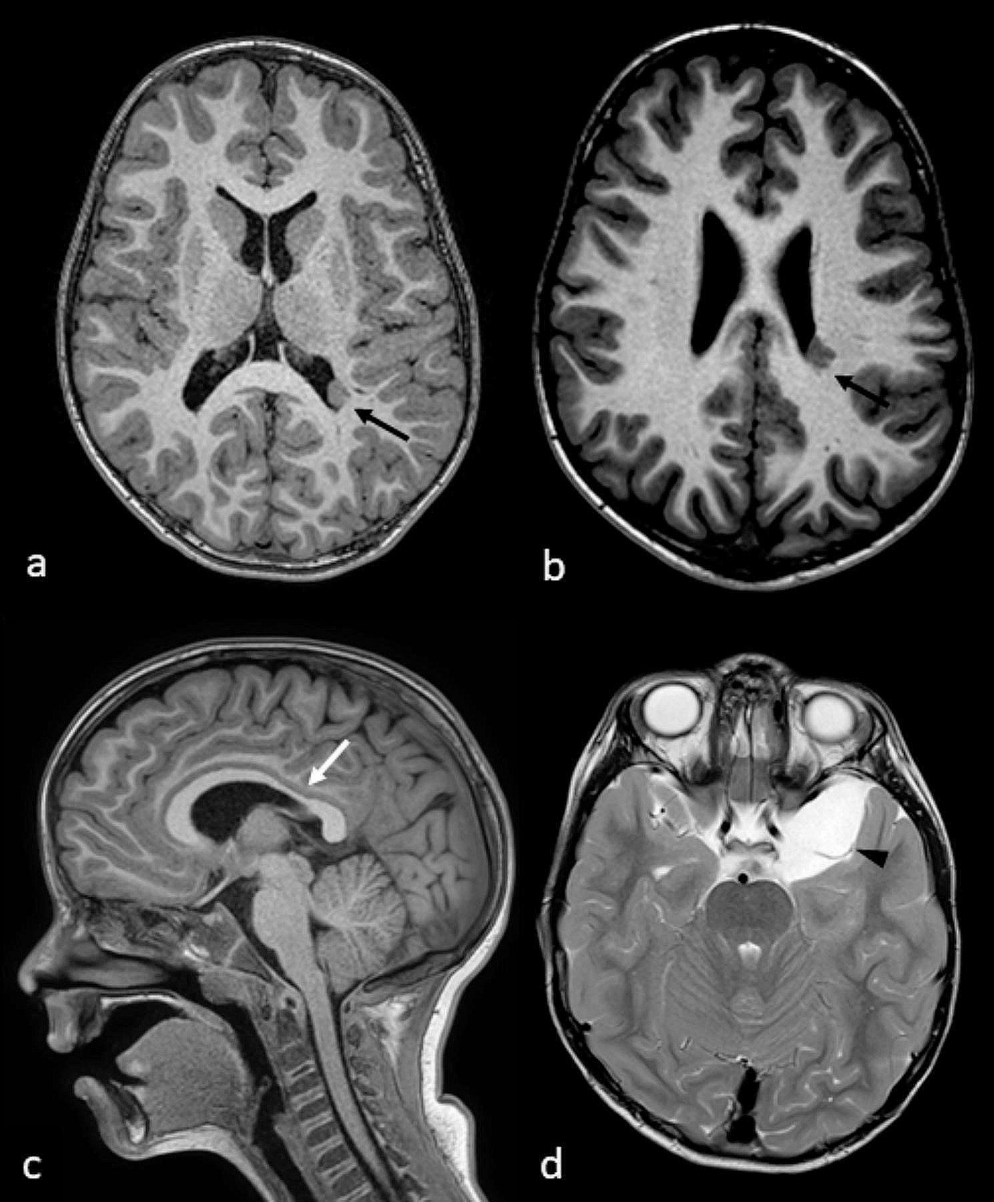
Brain MRI (6y 11 m), axial T1 (**a**-**b**), midline sagittal T1 (**c**) and axial T2 (**d**): periventricular nodular heterotopia in the posterior horn (a, black arrow) and middle portion (*b*, black arrow) of the left lateral ventricle, mild thinning of the corpus callosum istmo (*c*, white arrow), and arachnoid cyst in left mesial temporal lobe (*d*, arrowheads)

Brain MRI performed at 2y11m (last follow-up 6y11m) revealed two periventricular nodules, isointense to grey matter on all sequences, in the lateral wall of posterior horn and middle portion of the left lateral ventricle, suggestive of periventricular nodular heterotopia (PVNH) [Fig. 2a-b ]. A mild thinning of the corpus callosum (istmo) [Fig. 2c] and a left mesial temporal arachnoid cyst [Fig. 2d] were also present.

Array-CGH revealed a segmental duplication of 250Kb on the Y-chromosome: arr[GRCh37] Yq11.222(21058897_21311923)x2. The father was unavailable for segregation analysis.

WES revealed a novel heterozygous nonsense variant in the *TAOK1* gene: NM_020791.4:c.[1414 C > T];[=], NP_065842.1:p.[(Arg472*)];[=]. The variant was absent in the mother, while the father was unavailable for testing.

## Discussion and conclusions

We report a 5-year-old child with NDD, macrocephaly, mildly dysmorphic facial features and PVNH. WES identified the heterozygous nonsense variant NM_020791.4:c.1414 C > T in the *TAOK1* gene, which has not been previously described in the literature or in databases (ClinVar, LOVD; accessed on 2023/09/14). The variant is absent in the reference population databases gnomAD v2.1.1/v3.1.2 and 1000 Genomes project (accessed on 2023/09/14). It generates a premature stop codon in exon 14 out of 20, which is expected to result in nonsense-mediated decay of the transcript, and haploinsufficiency is already known to be consistent with the molecular mechanism of the disease [11, 13]. We could not confirm whether it occurred *de novo*: the variant was not present in the mother, while the father was unavailable for testing and his clinical records were not accessible. However, inheritance from a mildly affected parent has been described in five cases [14, 16], suggesting the possibility of incomplete penetrance or variable expressivity. Based on the recommendations of the American College of Medical Genetics [19] and on the clinical similarities with published cases of *TAOK1*-related NDD [Table 1, Supplementary Materials], the variant was classified as likely pathogenic (criteria PVS1, PM2) and considered responsible for the proband’s phenotype. The 250Kb duplication detected in Yq11.222 occurs in a region that contains only a redundant paralogue of chromosome 6 gene *CD24*. The Database of Genomic Variants (DGV, accessed on 2023/09/14) contains a few duplications spanning or overlapping this region, while comparable and larger duplications are reported as benign/likely benign on ClinVar (accessed on 2023/09/14). Therefore, although the father was unavailable for segregation analysis, this variant was considered unrelated to the proband’s phenotype.

**Table 1:** overview of clinical, genetic and brain MRI’s data of TAOK1-related NDDs reported patients (including our clinical report). Extensive information are in Supplementary Materials. *Abbreviations* SD = Standard Deviations; ADHD = Attention Deficit Hyperactivity Disorder; ASD = Autism Spectrum Disorder; PVNH = Periventricular Nodular Heterotopia; CC = corpus callosum. *Notes (****a****)* = patient N #23 reported by Van Woerden et al. 2021 is originally described by Xie et al. 2016 (Xie et al., 2016); *(****b****)* = Macrocephaly is defined as OFC **≥** 2 SD; *(****c****)* = “global developmental delay” refers to a delay in two or more developmental domains (gross motor/fine motor, cognitive, speech/language, personal/social, activities of daily living) in children younger than 5 years of age; *(****d****)* = “autism and autistic features” refers either to children with established diagnosis of ASD and children with mild autistic traits that do not satisfy the diagnostic criteria for ASD by standard scales

Paper	van Woerden 2021^(a)^	Dulovic-Mahlow 2019	Hunter 2022	Wang 2023	Basel-Salmon 2021	OUR PATIENT	TOTAL
***Gender***	males: 14/23	males: 5/8	males 2/4	male	female	male	males: 23/38 **(58%)**
***Type of mutation***	frameshift 5/23 splicing 3/23 missense 5/23 nonsense 6/23 exon deletion 1/23 large deletion 3/23	frameshift 1/8 missense 4/8 nonsense 3/8	frameshift 2/4 splicing 1/4 missense 1/4	frameshift	splicing	nonsense	frameshift 9/38 splicing 5/38 missense 10/38 nonsense 10/38 large deletion 3/38 exon deletion 1/38
***Inheritance***	de novo 16/19 inherited 3/19	de novo 8/8	de novo 2/4 inherited 2/4	De novo	De novo	N/A	de novo: 28/33 **(84%)** inherited: 5/33
***Brain MRI***	normal 11/17 unspecific findings 6/17 (arachnoid cyst, incomplete hippocampal inversion, hydrocephalus, thinning of CC, ventriculomegaly, Chiari I malformation, delayed myelination).	1/1 “leukodystrophy”	4/4 unspecific findings (arachnoid cyst, ventriculomegaly, mild bilateral parietal volume loss, multifocal subcortical gliosis consistent with perinatal injury)	N/A	N/A	PVNH, arachnoid cyst, thinning of CC	11/23: normal **(48%)** 11/23 unspecific findings 1/23: PVNH (+ unspecific findings)
***Dysmorphic facial features***	9/9 (mostly high forehead, downslanting palpebral fissures, bulbous nose, micrognathia)	6/8 (mostly large or high forehead, downslanting palpebral fissures, low set ears).	3/4 (mostly prominent forehead)	N/A	frontal bossing	high forehead, long and pronounced philtrum, bulbous nose, uplifted earlobes	20/23 **(87%)**
***Macrocephaly*** ^***(b)***^	8/20	3/8	3/4	Yes	Yes	Yes	16/35 **(46%)**
***Global developmental delay*** ^***(c)***^	18/20	6/8	4/4	N/A	No	Yes	29/34 **(85%)**
***Intellectual disability***	15/21	4/8	2/2	No	No	No	21/34**(69%)**
***ADHD or attention issues***	4/20	2/8	1/3	N/A	No	No	7/33 **(21%)**
***ASD or autistic features*** ^***(d)***^	5/17	2/8	2/3	Yes	No	Yes	11/31 **(35%)**
***Hypotonia***	12/21	6/8	4/4	N/A	No	No	22/35 **(63%)**
***Joint hypermobility***	7/21	2/8	3/3	N/A	N/A	No	12/33 **(36%)**
***Seizures***	2/2	1/8	1/4 (complex febrile seizures)	N/A	N/A	No	4/15 **(27%)**
***Eyes/visual problems***	7/19 strabismus, refraction’s abnormality, cataracts	1/8	2/4 (strabismus, ptosis, refraction’s abnormality)	N/A	N/A	acquired left-eye esophoria	11/32 **(34%)**
**History of feeding difficulties**	11/20 (swallowing difficulties, reflux, drooling)	1/1 (swallowing and oral motor difficulties)	3/4	N/A	N/A	No	15/26 **(58%)**

In order to better elucidate the phenotypic spectrum of *TAOK1*-related NDD, we have summarized the available data from 37 previously reported individuals with NDD [Table 1, Supplementary Materials] [11–16]. A few cases included in large cohort studies have insufficient clinical description and were not considered in this report [9, 10, 15, 20, 21]. A recent report of a de novo frameshift variant in a girl with isolated childhood-onset tremor was also excluded, because, based on the available clinical data, she did not properly fit into the definition of NDD [22]. Prenatal cases were also excluded [17, 18].

The main clinical features resulting from this review are developmental delay affecting speech and/or motor development, variable intellectual disability (ID), ASD, behavioral abnormalities, macrocephaly, hypotonia, joint hypermobility and dysmorphic facial traits (high forehead, downslanted palpebral fissures, low-set ears, bulbous nose and micrognathia). Less frequently reported are also feeding problems and limb undergrowth, and four patients had seizures [11, 12, 14, 16].

Our proband’s phenotype, characterized by severe language delay, poor communicative skills, acquired macrocephaly and mild dysmorphic facial features, is consistent with this clinical spectrum.

Neuroimaging data are available for only 22 patients with *TAOK1*-related NDDs [Dulovic-Mahlow et al., 2019; van Woerden et al., 2021; Hunter et al., 2022]. In 11 patients (50%) brain MRI was reported as normal, while the remaining had nonspecific, heterogeneous features, apparently not evocative of neuronal migration defects [Table 1, Supplementary Materials].

Our patient’s brain MRI displayed a mild thinning of the corpus callosum [Fig. 2c] and a left temporopolar arachnoid cyst [Fig. 2d]. Individually, similar nonspecific anomalies have already been observed [14, 16]. Interestingly, our patient also presented with PVNH in the left lateral ventricle [Fig. 2a-b].

PVNH is a congenital brain malformation of cortical development characterized by nodules of grey matter lining the ventricles, resulting from defects in the radial migration of cortical neurons along glial fibers during early brain development. It can be classified based on morphology, symmetry and location of heterotopic nodules along ventricle profiles on brain MRI images. Heterotopic nodules are highly epileptogenic, and may be isolated or associated with other brain abnormalities, variable intellectual disability and dysmorphic features [23, 24]. PVNH is also a genetically heterogeneous condition. Bilateral symmetrical multiple PVNH, often associated with cardiac malformations, is mostly related to defects in the *FLNA* gene (classical X-linked PVNH). Less typical PVNH forms, represented by bilateral asymmetrical and unilateral PVNH, may be caused by biallelic variants in *ARFGEF2*, *DCHS1*, *MCPH1, INTS8, FAT4* or heterozygous defects in *ERMARD*, *NEDD4L* and *MAP1B*, as well as several chromosomal abnormalities. Most of these genes are involved in microtubule regulation, vesicle trafficking, cell-cell adhesion and cell polarity. A cumulative dosage effect of multiple genes in the pathogenesis of non-classical PVNH has been hypothesized [25–27].

Yu et al. recently reported one case of brain MRI abnormalities in a fetus with a *de novo* missense *TAOK1* variant, in which bilateral polymicrogyria of the lateral fissure area could not be ruled out by the authors [18]. It would be interesting to know whether a postnatal MRI would confirm a malformation of cortical development.

The finding cortical migration defects such as of PVNH is highly consistent with the cellular and developmental role of *TAOK1* in neuronal migration, as defined by previous functional studies both in vitro and in animal models [4, 11, 13, 14].

In the embryonic mouse brain, Van Woerden and colleagues [14] observed that reduced *TAOK1* expression levels affected neural migration in vivo. Through in utero electroporation, they performed shRNA-mediated knockdown of *Taok1* (*Taok1*^kd^) at embryonic day 14.5, a critical time for neurodevelopment, and observed a significant deficit in the migration of neurons derived from the transfected progenitors, at birth and at postnatal day 7.

Similarly, by comparing *Taok1*^*+/−*^ (haploinsufficient) and WT mice, Wang et al. [13] detected a significant decrease in neuron density in the upper layer of the caudal cortex, confirming a defect in neuronal migration.

To the best of our knowledge, this is the first report of PVNH in an individual with *TAOK1*-related NDD, suggesting a possible expansion of the phenotype of *TAOK1*-of this condition.

Although further evidence is needed, this finding could represent a confirmation in humans of the essential role of *TAOK1* in neuronal migration, as already indicated by functional studies in vitro and in vivo.

This work also highlights the importance of including brain MRI in the diagnostic workup of affected individuals, even in the absence of epilepsy or macrocephaly.

### Electronic supplementary material

Below is the link to the electronic supplementary material.

Supplementary Material 1

Supplementary Material 2

## Data Availability

Data and materials are available from the corresponding author upon reasonable request.

## Abbreviations

ASD: autism spectrum disorder
MRI: magnetic resonance imaging
NDD/NDDs: neurodevelopmental disorder/s
PVNH: periventricular nodular heterotopia
*TAOK1*: Thousand and one aminoacid kinase 1
WES: whole exome sequencing
