# Supplementary material for: Heterozygous truncating variant of TAOK1 in a boy with periventricular nodular heterotopia: a case report and literature review of TAOK1-related neurodevelopmental disorders
Source: BMC Med Genomics. 2024 Mar 5;17:68. doi: 10.1186/s12920-024-01840-8 (PMC10916022; doi:10.1186/s12920-024-01840-8)
Supplement: Supplementary file 2 — Supplementary Material 2 [file 12920_2024_1840_MOESM2_ESM.pdf]

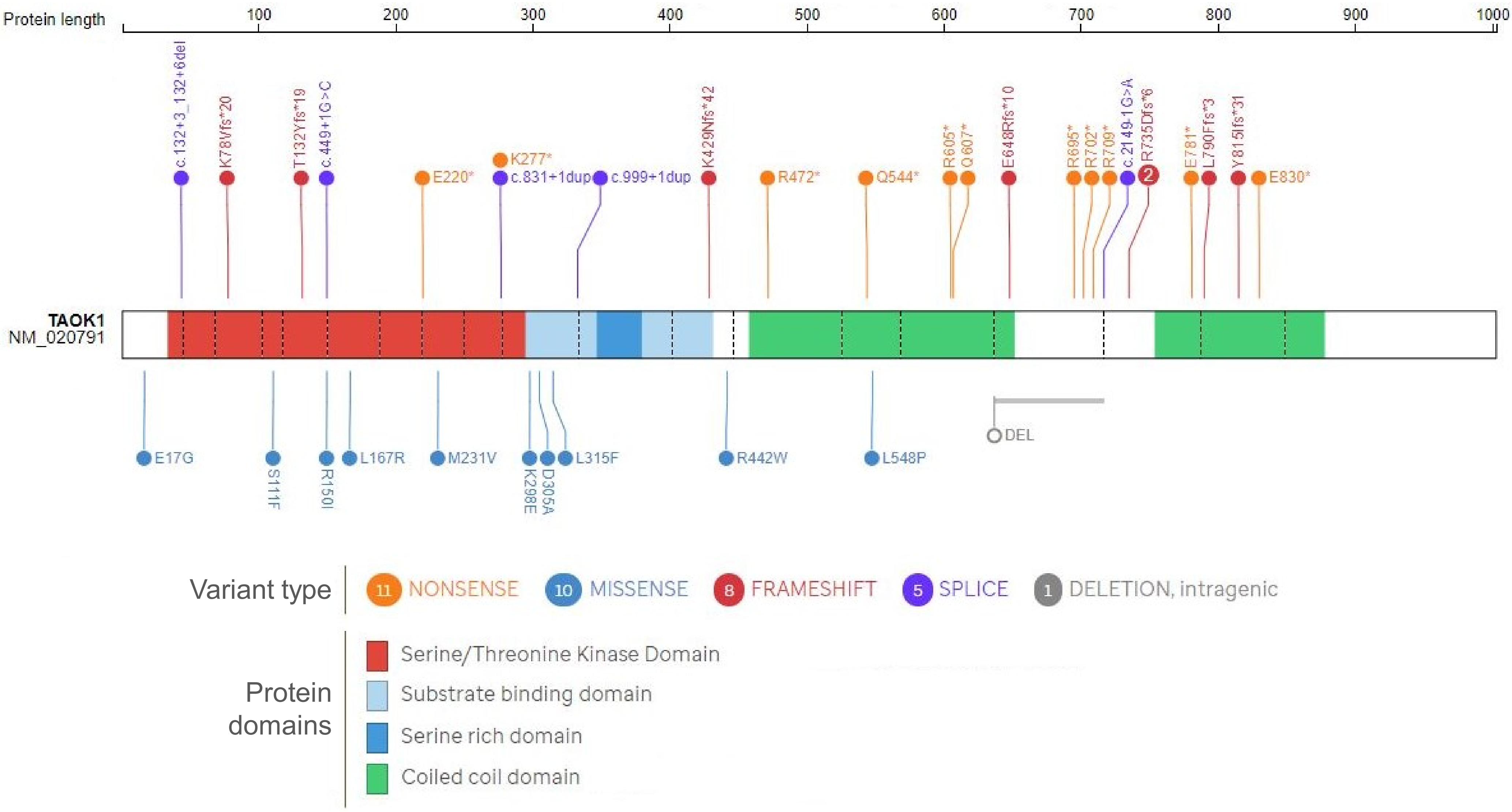

Supplementary Figure 1 – Scheme representing the TAOK1 protein and the variants from individuals with TAOK1-related disease (from Supplementary Table 1). Three whole gene deletions were not shown. Dashed vertical lines represent exon-exon boundaries. Graphics made with ProteinPaint (https://www.nature.com/articles/ng.3466).
